# Effect of semi-quantitative procalcitonin assay on the adequacy of empirical antibiotics and mortality in septic patients

**DOI:** 10.1186/cc12915

**Published:** 2013-11-05

**Authors:** Dana Dharaniyadewi, Khie Chen Lie, Nanang Sukmana, C Martin Rumende

**Affiliations:** 1 Department of Internal Medicine, Faculty of Medicine, Universitas Indonesia, Jakarta, Indonesia; 2 Division of Tropical Medicine and Infectious Diseases, Department of Internal Medicine, Faculty of Medicine, Universitas Indonesia, Jakarta, Indonesia; 3 Division of Allergy and Clinical Imunology, Department of Internal Medicine, Faculty of Medicine, Universitas Indonesia, Jakarta, Indonesia; 4 Division of Respirology and Critical Care, Department of Internal Medicine, Faculty of Medicine, Universitas Indonesia, Jakarta, Indonesia

## Background

Sepsis is a serious clinical condition with a considerable morbidity and mortality. Procalcitonin (PCT) is a good biomarker for early diagnosis and infection monitoring. A semi-quantitative PCT assay can be performed at the bedside and has good diagnostic value [1,2]. The present study aimed to investigate the effect of a semi-quantitative PCT test on the empirical antibiotic initiation time, the appropriateness of empirical antibiotics and mortality in septic patients.

## Materials and methods

The study design was a randomized diagnostic trial, which was also a pragmatic trial. Septic patients more than 18 years old with and without signs of organ hypoperfusion or dysfunction who were admitted to Cipto Mangunkusomo Hospital emergency department in the internal medicine unit were eligible. Subjects were randomly assigned to either a semi-quantitative PCT-examined group (study group) or a control group. Semi-quantitative PCT test results will be informed to the physicians taking care of the patients. The primary outcome was 14-day mortality. Secondary outcomes were the time of initiation and appropriateness of empirical antibiotics. A Tropical Infection Consultant will assess the appropriateness of empirical antibiotics based on Pedoman Umum Penggunaan Antibiotik Departemen Kesehatan Republik Indonesia.

## Results

**Figure 1:**
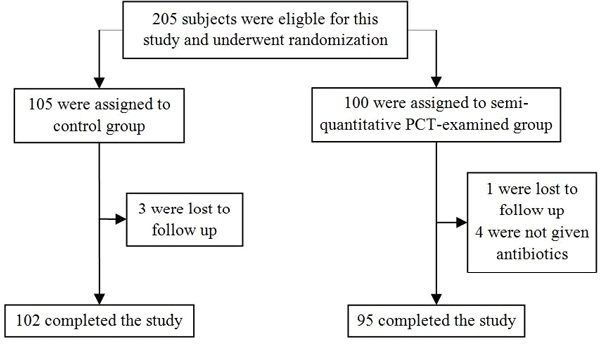
**Enrollment of patients and completion of the study**.

Two hundred and five patients met the inclusion criteria. Ninety-five of 100 subjects from the study group and 102 of 105 subjects from the control group were included in the analysis (Figure 1). Both groups have equal baseline characteristics (Table 1). The mortality risk was lower in the study group (RR 0.53; 95% CI 0.36 to 0.77). The study group had greater probability to have a first dose of empirical antibiotic in less than 6 hours compared with the control group (RR 2.48; 95% CI 1.88 to 3.26). No effect was seen in appropriateness of empirical antibiotics between groups (RR 0.99; 95% CI 0.92 to 1.08) (Table 2).

**Table 1:** Baseline characteristics of the patients

Characteristic	Semi-quantitative PCT-examined group, *n (%)*	Control group, *n *(%)
Age		
>60 years	28 (29.5)	23 (22.5)
≤60 years	67 (70.5)	79 (77.5)
Mean age (years)	51.4 ± 15.7	48.6 ± 46.0
Sex		
Male	42 (44.2)	40 (39.2)
Female	53 (55.8)	62 (60.8)
Sepsis severity		
Sepsis	57 (60.0)	54 (52.9)
Severe sepsis and septic shock	38 (40.0)	48 (47.1)
Comorbidity		
Without comorbidities	20 (21.1)	20 (19.6)
With comorbidities	75 (78.9)	82 (80.4)
Source of infection		
One source	82 (86.3)	86 (84.3)
≥2 sources	13 (13.7)	16 (15.7)
14-day mortality	26 (27.4)	53 (52.0)

**Table 2:** Effect of semi-quantitative procalcitonin assay on adequacy of empirical antibiotics and mortality in septic patients

Outcomes	Semi-quantitative PCT assay, *n *(%)		RR (95% CI)	*P *value
			
	Examined	Not examined		
Empirical antibiotic initiation time				
≤6 hours	83 (87.4)	36 (35.3)	2.48 (1.88 to 3.26)	<0.001
>6 hours	12 (12.6)	66 (64.7)		
Appropriateness of empirical antibiotics				
Appropriate	88 (92.6)	95 (93.1)	0.99 (0.92 to 1.08)	0.890
Inappropriate	7 (7.4)	7 (6.9)		
14-day mortality				
Yes	26 (27.4)	53 (52.0)	0.53 (0.36 to 0.77)	<0.001
No	69 (72.6)	49 (48.0)		

## Conclusions

Semi-quantitative PCT examination affects the empirical antibiotic initiation time and mortality in septic patients, but not the appropriateness of empirical antibiotics.
